# Expression of Selected Genes and Circulating microRNAs in Patients with Celiac Disease

**DOI:** 10.3390/medicina58020180

**Published:** 2022-01-25

**Authors:** Elena Maria Domsa, Ioana Berindan-Neagoe, Livia Budisan, Cornelia Braicu, Ioana Para, Alina Ioana Tantau, Olga Hilda Orasan, Lidia Ciobanu, Teodora Atena Pop, Gabriela Adriana Filip, Nicoleta Leach, Vasile Negrean, Daniela Matei, Vasile Andreica

**Affiliations:** 1 4th Medical Clinic, Department 5-Internal Medicine, Faculty of Medicine, “Iuliu Hatieganu” University of Medicine and Pharmacy, 400015 Cluj-Napoca, Romania; elennmary@yahoo.com (E.M.D.); alitantau@gmail.com (A.I.T.); olgaorasan@yahoo.com (O.H.O.); nicoleta_leach@yahoo.com (N.L.); vasile.negrean@umfcluj.ro (V.N.); 2 Research Center for Functional Genomics, Biomedicine and Translational Medicine, Faculty of Medicine, “Iuliu Hatieganu” University of Medicine and Pharmacy, 400337 Cluj-Napoca, Romania; ioananeagoe29@gmail.com (I.B.-N.); lbudisan@yahoo.com (L.B.); braicucornelia@yahoo.com (C.B.); 3 MedFUTURE, Research Center for Advanced Medicine, “Iuliu Hatieganu” University of Medicine and Pharmacy, 400337 Cluj-Napoca, Romania; 4 Department of Functional Genomics and Experimental Pathology, The Oncology Institute “Prof. Dr. Ion Chiricuta”, 400015 Cluj-Napoca, Romania; 5 3rd Medical Clinic, Department 5-Internal Medicine, Faculty of Medicine, “Iuliu Hatieganu” University of Medicine and Pharmacy, 400162 Cluj-Napoca, Romania; ciobanulidia@yahoo.com (L.C.); teodorapop@gmail.com (T.A.P.); dmatei68@gmail.com (D.M.); andreicav@umfcluj.ro (V.A.); 6 Department of Physiology, Faculty of Medicine, “Iuliu Hatieganu” University of Medicine and Pharmacy, 400006 Cluj-Napoca, Romania; adrianafilip33@yahoo.com

**Keywords:** celiac disease, circulating microRNAs, gene expression, biomarkers, networks

## Abstract

*Background and Objectives*: Celiac disease (CD) is an immune-mediated enteropathy with characteristic intestinal alterations. CD occurs as a chronic inflammation secondary to gluten sensitivity in genetically susceptible individuals. Until now, the exact cause of the disease has not been established, which is why new studies have appeared that address the involvement of various genes and microRNAs (miRNAs) in the pathogenesis. The aim of the study is to describe the expression of selected genes (Wnt family member 3, *WNT3*; Wnt family member 11, *WNT11*; tumor necrosis factor alpha, *TNFα*; mitogen-activated protein kinase 1, *MAPK1*; AKT serine/threonine kinase 3, *AKT3*; phosphatidylinositol-4,5-bisphosphate 3-kinase catalytic subunit alpha, *PIK3CA*; and cyclin *D1, CCND1*) and miRNAs (miR-192-5p, miR-194-5p, miR-449a and miR-638) in adult patients with CD. *Materials and Methods*: In total, 15 patients with CD at diagnosis (newly diagnosed), 33 patients on a gluten-free diet (GFD) for at least 1 year and 10 controls (control) were prospectively included. Blood samples were evaluated by quantitative real-time polymerase chain reaction (qRT-PCR). *Results*: The results show that *TNFα*, *MAPK1* and *CCND1* were significantly overexpressed (*p* = 0.0249, *p* = 0.0019 and *p* = 0.0275, respectively) when comparing the newly diagnosed group to the controls. The other genes studied in CD patients were mostly with high values compared to controls, without reaching statistical significance. Among the miRNAs, the closest to a statistically significant value was miR-194-5p when the newly diagnosed group versus control (*p* = 0.0510) and GFD group versus control (*p* = 0.0671) were compared. The DIANA and miRNet databases identified significant functional activity for miR-449a and miR-192-5p and an interconnection of miR-194-5p and miR-449a with *CCND1*. *Conclusions*: In conclusion, genes and circulating miRNAs require further studies as they could represent important biomarkers in clinical practice.

## 1. Introduction

Celiac disease (CD) is an immune-mediated enteropathy that occurs in genetically predisposed persons, being caused by the ingestion of gluten [1,2]. It is more common in women and can occur at any age. Although the prevalence of the disease has increased in last decades and is present in around 0.5–1% of the general population, many cases remain undiagnosed [2,3]. Clinically, the disease may present digestive manifestations, such as diarrhea, abdominal pain, involuntary weight loss, etc., and/or extra-digestive signs, such as fatigue, anemia, high transaminases levels, vitamin deficiencies, etc. [3]. CD in adults is confirmed if positive serological tests (IgA anti-transglutaminase antibodies, IgA tTG; and/or IgA anti-endomysium antibodies, IgA EMA) are accompanied by characteristic histological changes (villous atrophy, increased intraepithelial lymphocytes and crypt hyperplasia) [1]. Even though various drugs have been studied so far, the only treatment accepted is the gluten-free diet (GFD) [1,4]. Practically, the cause of the disease has not been established exactly to this date and, in this sense, various studies have investigated involvement of microRNAs in its pathogenesis [3].

MicroRNAs (miRNAs) are small non-coding single-chain RNAs (ribonucleic acids) that impede the translation of various mRNAs (messenger RNAs), thereby suppressing their target genes. Due to their role in the diagnosis and treatment of several digestive diseases, including celiac disease, miRNAs could represent a class of promising biomarkers; thus, they could help us to better understand the pathogenetic mechanisms of the disease [1].

Bioinformatics analyses have been described in the literature where new genes and pathways of miR-192, miR-194-5p, miR-449a and miR-638 with a role in pathogenesis have been identified. Thus, the 3-phosphoinositide dependent protein kinase 1 *(PDPK1), KIT* ligand *(KITLG), MET* proto-oncogene, receptor tyrosine kinase *(MET), AKT* serine/threonine kinase 2 *(AKT2),* colony stimulating factor 1 receptor *(CSF1R),* collagen type IV alpha 4 chain *(COL4A4), CCND1*, fibroblast growth factor 23 *(FGF23),* integrin subunit beta 8 *(ITGB8),* fibroblast growth factor 2 *(FGF2),* laminin subunit gamma 1 *(LAMC1),* protein kinase C alpha *(PRKCA),* retinoid X receptor alpha *(RXRA),* platelet derived growth factor receptor alpha *(PDGFRA)* and vascular endothelial growth factor A *(VEGFA)* genes targeted by miR-449a through the phosphoinositide-3-kinase-serine/threonine-protein kinase (PI3K-Akt) signaling pathway were described. In addition, bioinformatics predictions have shown that *CREB* binding protein *(CREBBP),* transglutaminase 2 *(TGM2), APC* regulator of WNT signaling pathway 2 *(APC2),* frizzled class receptor 4 *(FZD4), CCND1* and transcription factor 7 *(TCF7)* have an important role in the Wnt signaling pathway and are targets of the overexpressed miR-638; further, the *MAPK1,* fibroblast growth factor receptor 1 *(FGFR1),* protein tyrosine phosphatase receptor type J *(PTPRJ),* insulin receptor *(INSR),* sorbin and SH3 domain containing 1 *(SORBS1*), tight junction protein 1 *(TJP1),* transforming growth factor beta receptor 1 *(TGFBR1)* and *TCF7* genes implicated in the adherens junction pathways are targeted by miR-192, which is downregulated in patients with CD. Another miRNA studied in CD whose expression is reduced, miR-194-5p, has, as targeted genes, *CRK* proto-oncogene, adaptor protein *(CRK), PDPK1, RAP1B,* member of RAS oncogene family *(RAP1B), AKT2,* Rho GTPase activating protein 5 *(ARHGAP5),* cyclin *D2 (CCND2),* X-linked inhibitor of apoptosis *(XIAP), (LAMC1), MAPK1,* diaphanous related formin 1 *(DIAPH1),* protein phosphatase 1 catalytic subunit beta *(PPP1CB), ITGB8* and *PRKCA*, with a role in focal adhesion [3,5,6,7].

The genetic component of CD has long been studied, so far, with various methods, such as candidate gene sequencing, exome sequencing, single-nucleotide polymorphism (SNP) genotyping and epigenetic screening. Thus, by associating gene expression evaluation with protein–protein interactions (PPIs) and pathway analysis, we would have a deeper picture of CD development. Key pathways related to potential biological processes, such as dysregulated immune system events, reduced regulated cell division and altered absorption, have been identified. These pathways have been associated with villous atrophy and a high level of intraepithelial lymphocytes (IELs). All this information could provide important data in the future regarding the discovery of biomarkers useful in the prognosis, diagnosis and treatment of CD [8,9]. The role of the Wnt pathway in the gastrointestinal tract is to preserve the self-renewal capacity of epithelial stem cells. In addition, this pathway requires several feedback loops that stabilize the opposite processes of cell proliferation and differentiation. Several studies have shown that the mutations involved in the aberrant activation of the Wnt pathway cause the spread of undifferentiated progenitors and the appearance of cancer [10]. Innate lymphoid cells have the capacity to produce proinflammatory cytokines, including *TNF-α*, involved in the tissue damage in CD [11]. *MAPK1* has a role in cellular and signaling processes such as proliferation, differentiation, transcriptional regulation and development [12]. MAPK pathways are involved in inflammation and cancer [13]. Rostami-Nejad et al. performed a network analysis and described five central nodes, including *PIK3CA* and eight significant differentially expressed genes involved in CD pathogenesis, with a possible role in prognosis and treatment [14]. *AKT3*, also called AKT kinase, is one of the three closely related serine/threonine-protein kinases (*AKT* serine/threonine kinase 1, *AKT1; AKT2* and *AKT3*) implicated in CD pathogenesis. *AKT3* is involved in a number of processes such as metabolism, proliferation, cell survival, growth and angiogenesis. The association of *Akt* with the plasma membrane is related to the fact that *Akt* is a downstream mediator of the PI 3-K pathway [15,16]. A possible role of *AKT3* has been described by Zhang and Sun in the pathogenesis of ulcerative colitis [17]. *CCND1* is a protein involved in the regulation of the cell cycle. Overexpression of *CCND1* contributes to cell proliferation and the appearance of various types of tumors, leading to a poor prognosis [18].

Based on literature data, this study aims to evaluate the expression of the *WNT3, WNT11, TNFα, MAPK1, AKT3, PIK3CA* and *CCND1* genes and miR-192-5p, miR-194-5p, miR-449a and miR-638 in patients with CD in order to highlight potential correlations with clinical data and their importance as therapeutic targets.

## 2. Materials and Methods

### 2.1. Patients and Controls

Study participants were recruited from the “Prof. Dr. Octavian Fodor” Regional Institute of Gastroenterology and Hepatology and from the 4th Medical Clinic, Cluj-Napoca, between 2015 and 2018.

Our prospective, analytical, observational study included 58 subjects who were divided into 3 groups. Group 1 consisted of 15 patients with CD at diagnosis, on a gluten-containing diet (newly diagnosed); group 2 consisted of 33 patients on a GFD for at least 1 year (GFD); group 3 consisted of 10 controls (control). A comparative analysis was performed as follows: newly diagnosed versus control; GFD versus control; newly diagnosed versus GFD.

The diagnosis of celiac disease was made based on the patient anamnesis, physical examination, dosing of anti-transglutaminase IgA antibodies and/or anti-endomysium IgA antibodies, duodenal biopsies and their histological interpretation (all patients presented villous atrophy) and determination of HLA-DQ2 and HLA-DQ8 (in those with a discrepancy between serology and histology). Differential diagnoses with other diseases were also made and other causes of villous atrophy (such as IgA deficiency, common variable immunodeficiency syndrome, infectious, inflammatory diseases, the use of drugs, etc.) have been ruled out. At the time of diagnosis, all patients were on a gluten-containing diet.

The control group included subjects with dyspepsia, but with normal endoscopic and histological findings, with negative anti-transglutaminase IgA antibodies and/or anti-endomysium IgA antibodies, without chronic or autoimmune diseases. 

The study participants were evaluated either for diagnostic purposes (new CD cases), or for follow-up (GFD cases), or for validation of the control group (normal duodenal histology and negative antibodies). The aim of this study is to assess the level of genes and miRNAs expression in newly diagnosed patients compared with those on a GFD and with controls, to analyze whether there were differences between groups.

From all subjects, we collected blood samples that were processed to obtain serum, plasma and the leukocyte pellet (peripheral blood mononuclear cells—PBMC). The leukocyte pellet was introduced in a TriReagent solution and stored at −80 °C until use.

### 2.2. RNA Isolation and Quantitative Real-Time Polymerase Chain Reaction (qRT-PCR) Evaluation 

Total RNA from blood was extracted using TriReagent (Invitrogen Corporation, Carlsbad, CA, USA) based on the producer’s recommended protocol. RNA concentrations and quality were assessed using NanoDrop-1100. Sample purity was assessed based on the spectral data and purity ratios. For gene expression evaluation, total RNA samples were treated for complete digestion of DNA (deoxyribonucleic acid) with TURBO DNA-free™ Kit (Invitrogen) according to the manufacturer’s protocol. Total RNA (50 ng for miRNA expression and 1000 ng for gene expression/sample) was transcribed into cDNA (complementary DNA) using a TaqMan MicroRNA Reverse Transcription Kit (Applied Biosystems, Carlsbad, CA, USA) for miRNA expression analysis and a High Capacity cDNA Reverse Transcription Kit (Applied Biosystems, Carlsbad, CA, USA) for gene expression evaluation. For miRNA amplification, we used a TaqMan Fast Advanced Master Mix (Applied Biosystems) and a SYBR Select Master Mix (Applied Biosystems, Carlsbad, CA, USA) for gene expression. qRT-PCR was performed with a ViiA™ 7 System (Applied Biosystems, Carlsbad, CA, USA) in a 5 µL (miRNA amplification) and a 10 µL (gene amplification) volume using a 384-well plate. All the samples were evaluated in duplicate.

The primer sequences for gene expression protocol were as follows:

- *TNF alfa* left: 5′ AGCCCATGTTGTAGCAAACC 3′;
- *TNF alfa* right: 5′ TCTCAGCTCCACGCATT 3′;
- *WNT3* left: 5′ CCTGCAAGTAGGGCACCA 3′;
- *WNT3* right: 5′ CCCATGAGACTTCGCTGAAT 3′;
- *WNT11* left: 5′ AGCTCGCCCCAACTATT 3′;
- *WNT 11* right: 5′ ATACACGAAGGCCGACTCC 3′;
- *CCND1* left: 5′ GCTGTGCATCTACACCGACA 3′;
- *CCND1* right: 5′ TTGAGCTTGTTCACCAGGAG 3′;
- *PIK3CA* left: 5′ CGAGATCCTCTCTCTGAAATCAC3′;
- *PIK3CA* right: 5′ GAATTTCGGGGATAGTTACACAA 3′;
- *AKT3* left: 5′ TTGCTTTCAGGGCTCTTGAT 3′;
- *AKT3* right: 5′ CATAATTTCTTTTGCATCATCTGG 3′;
- *MAPK1* left: 5′ GTTCAGAACTACCCCCTGCTT 3′;
- *MAPK 1* right: 5′ CAGAGACGCAGAATGACTGG 3′.

As housekeeping, the following were used:

- Beta-2-microglobulin *(B2M)* left: 5′ CACCCCCACTGAAAAAGATGAG 3′;
- *B2M* right: 5′ CCTCCATGATGCTGCTTACATG 3′;
- Ribosomal protein, large, P0 *(RPLPO)* left: 5′ CCCAATTGTCCCCTTACCTT 3′;
- *RPLPO* right: 5′ ACCCAGCTCTGGAGAAGTCA3′;
- miR-194-5p, Life technology, cat No. 477956;
- miR-449a, Life technology, cat No. 478561;
- miR-192-5p, Life technology, cat No. 478262;
- miR-638, Life technology, cat No. 478187;
- U6 small nuclear RNA (U6), Life technology, cat No. 001973;
- Small nucleolar RNA, C/D box 48 (RNU48), Life technology, cat No. 001006.

### 2.3. Statistical Analysis

The statistical analyses were conducted using GraphPad Prism software (version 6.0; GraphPad Software, Inc., San Diego, CA, USA). Statistical significance was determined with the Student’s *t*-test. A probability (*p*) value of <0.05 was considered statistically significant.

DIANA-miRPath (http://www.microrna.gr/miRPathv3 (accessed on 5 May 2021)) is a widely used online bioinformatics tool that was also used in our study to determine various molecular functions for the case of the evaluated circular miRNA transcripts in CD versus normal controls. 

The integrated analysis for the altered miRNA and genes was performed using miRNet (https://www.mirnet.ca (accessed on 5 May 2021)), which is a valuable bioinformatics tool to generate miRNA-target interaction networks at different levels for a wide range of pathologies [19,20]. The miRNet network emphasizes a direct interconnection among the evaluated miRNAs and genes, displaying new insights in CD.

## 3. Results

### 3.1. General Characteristics of CD Patients and Controls

Subjects with newly diagnosed celiac disease were between 26 and 67 years old, those on a GFD were between 23 and 70 years old and controls were between 31 and 71 years old. 

The general characteristics of CD patients and controls are described in Table 1.

### 3.2. Gene Expression

**Figure 1:**
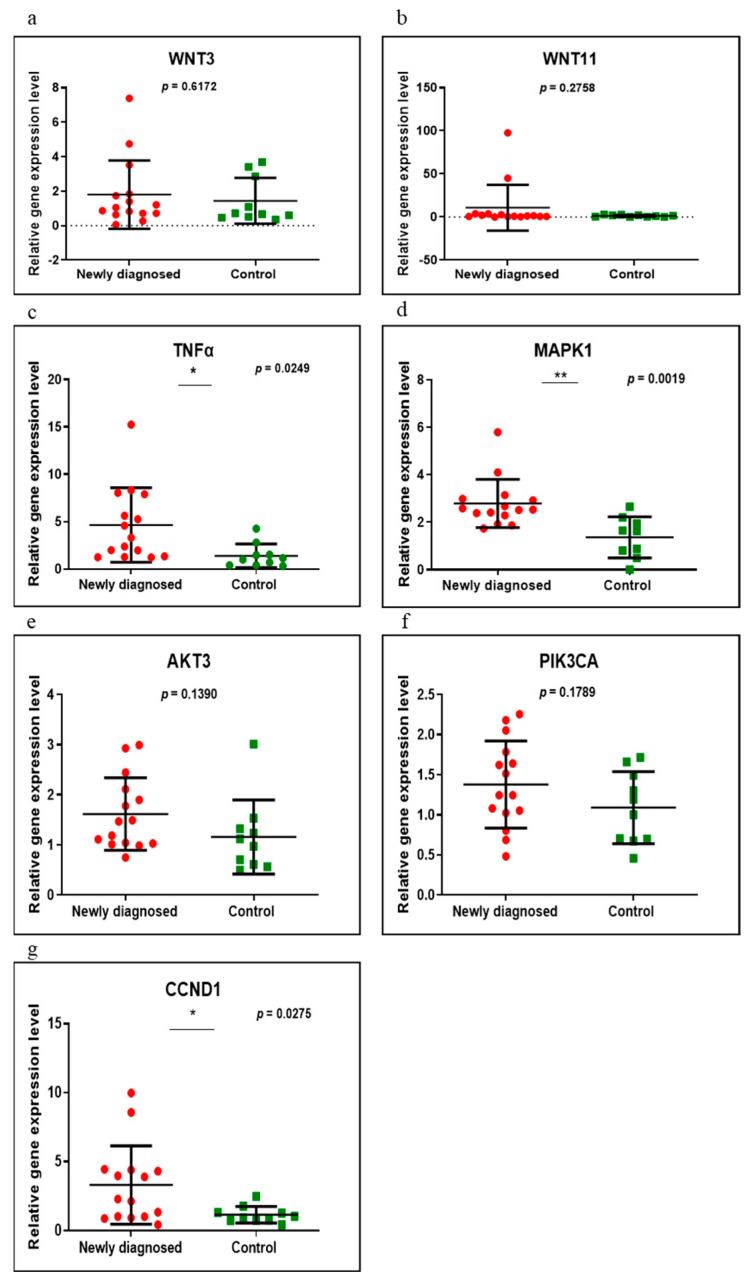
Expression levels of *WNT3* (**a**), *WNT11* (**b**), *TNFα* (**c**), *MAPK1* (**d**), *AKT3* (**e**), *PIK3CA* (**f**) and *CCND1* (**g**) in newly diagnosed CD group versus controls using qRT-PCR based on the TaqMan protocol. Abbreviations: * and ** *p* < 0.05.

**Figure 2:**
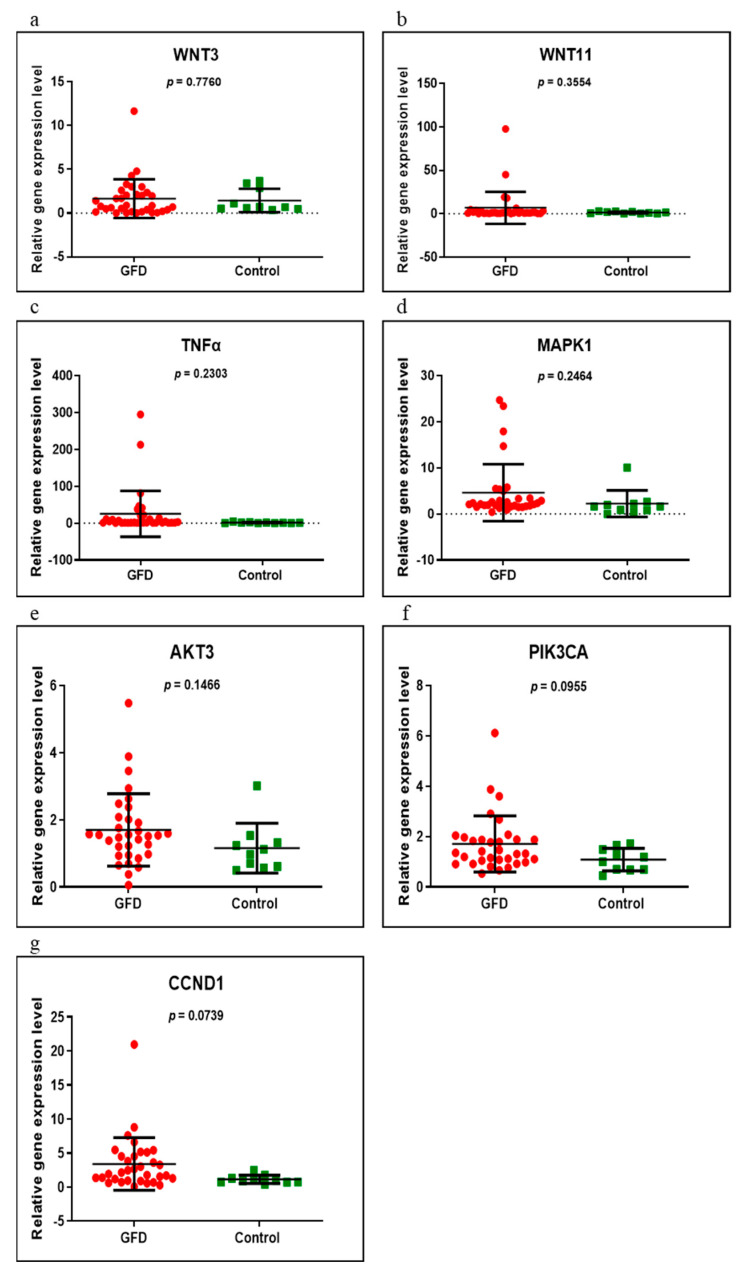
Expression levels of *WNT3* (**a**), *WNT11* (**b**), *TNFα* (**c**), *MAPK1* (**d**), *AKT3* (**e**), *PIK3CA* (**f**) and *CCND1* (**g**) in GFD CD group versus controls using qRT-PCR based on the TaqMan protocol.

**Figure 3:**
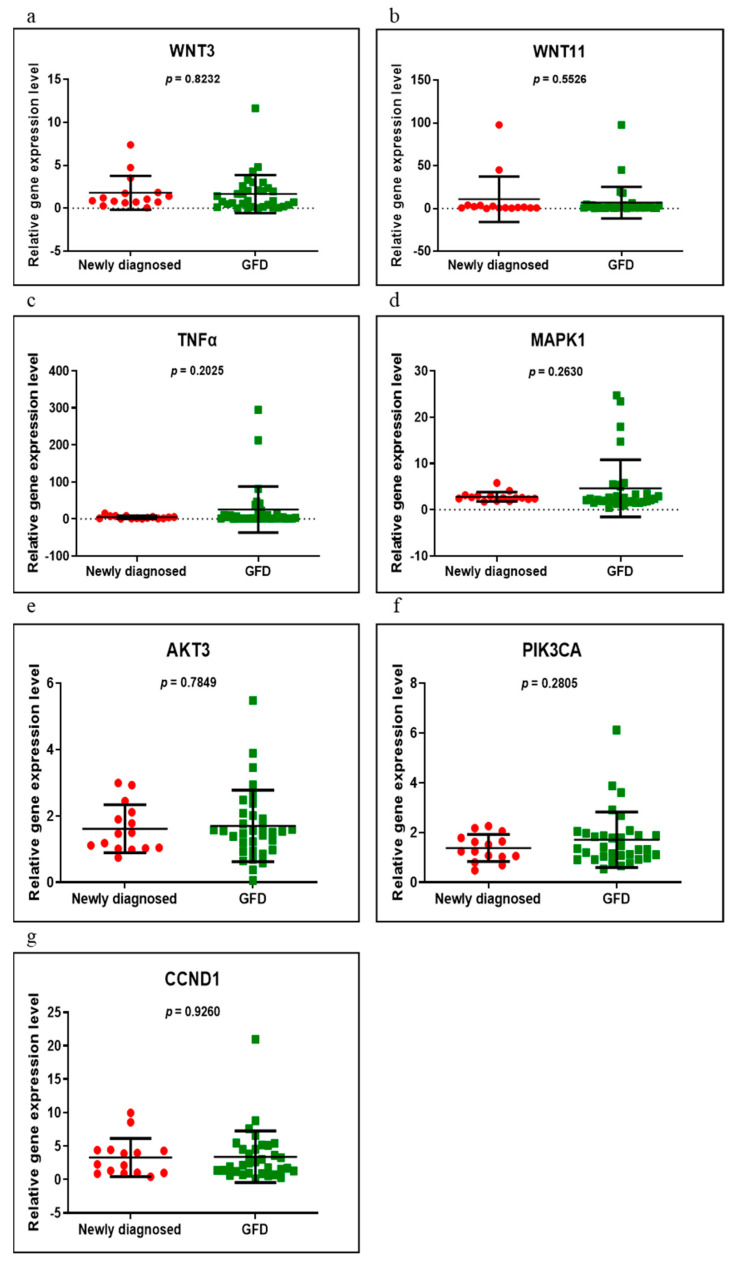
Expression levels of *WNT3* (**a**), *WNT11* (**b**), *TNFα* (**c**), *MAPK1* (**d**), *AKT3* (**e**), *PIK3CA* (**f**) and *CCND1* (**g**) in newly diagnosed CD versus GFD CD groups using qRT-PCR based on the TaqMan protocol.

Seven genes (*WNT3, WNT11, TNFα, MAPK1, AKT3, PIK3CA* and *CCND1*) were evaluated in the following groups of patients: newly diagnosed versus control, GFD versus control and newly diagnosed versus GFD. Of these, only three (*TNFα, MAPK1* and *CCND1*) were found to be significantly overexpressed when comparing the newly diagnosed group with control (*p* = 0.0249, *p* = 0.0019 and *p* = 0.0275, respectively) (Figure 1). In those on a GFD, compared to controls, all genes had an upward trend, but without reaching statistical significance (*p* > 0.05) (Figure 2). Comparing the newly diagnosed with the GFD groups, higher values were generally observed in those on a GFD but without significant differences (Figure 3).

### 3.3. miRNAs Expression

**Figure 4:**
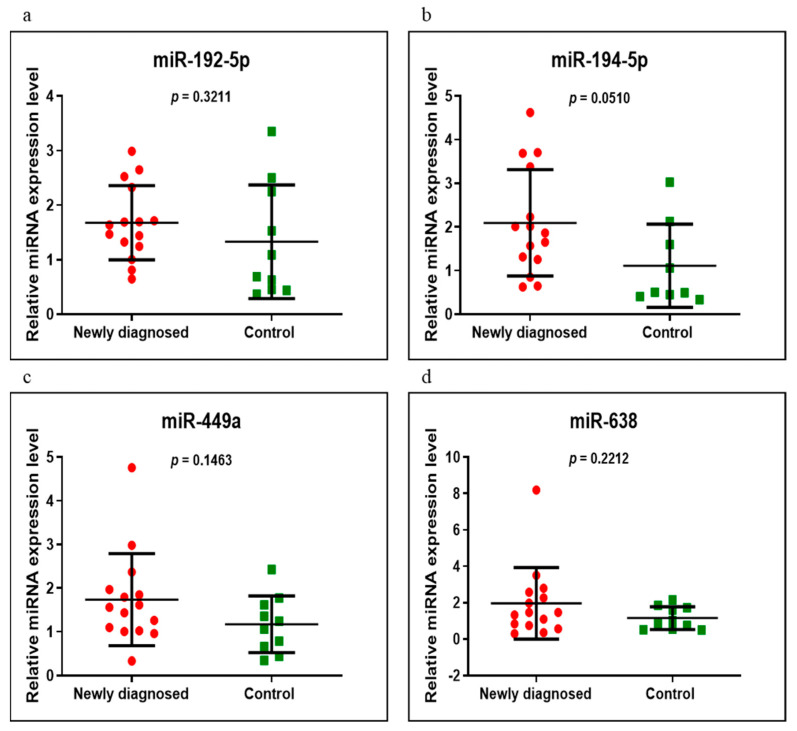
Expression levels of miR-192-5p (**a**), miR-194-5p (**b**), miR-449a (**c**) and miR-638 (**d**) in newly diagnosed CD group versus controls using qRT-PCR based on the TaqMan protocol.

**Figure 5:**
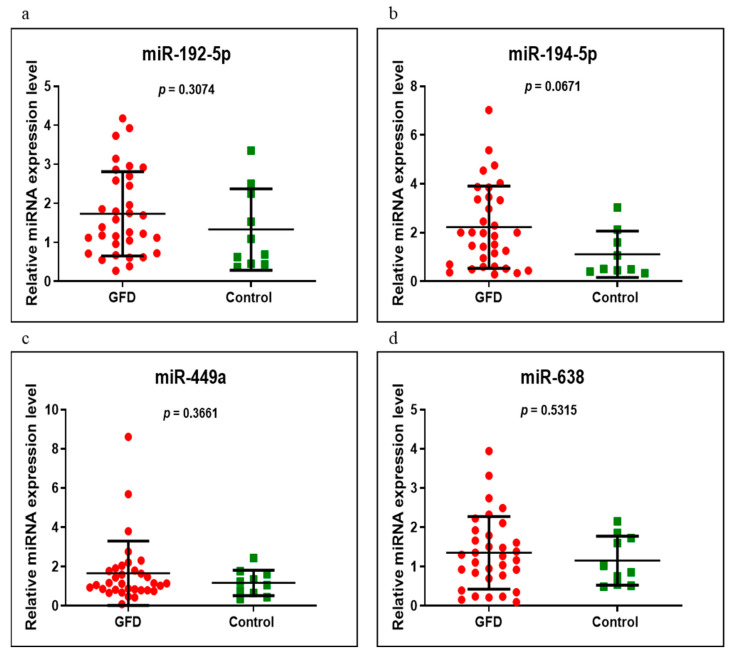
Expression levels of miR-192-5p (**a**), miR-194-5p (**b**), miR-449a (**c**) and miR-638 (**d**) in GFD CD group versus controls using qRT-PCR based on the TaqMan protocol.

**Figure 6:**
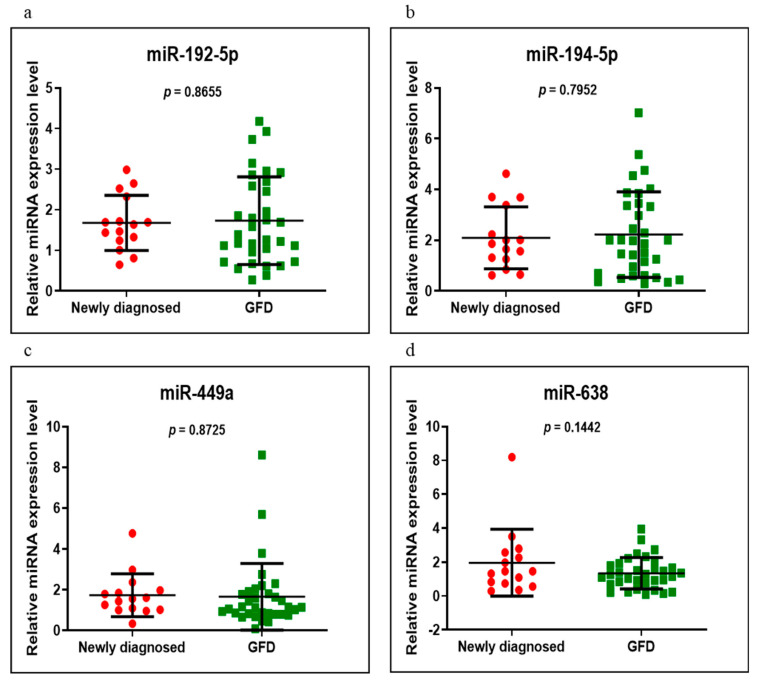
Expression levels of miR-192-5p (**a**), miR-194-5p (**b**), miR-449a (**c**) and miR-638 (**d**) in newly diagnosed CD versus GFD groups using qRT-PCR based on the TaqMan protocol.

miR-192-5p, miR-194-5p, miR-449a and miR-638 were analyzed on the same groups of patients, respectively, newly diagnosed versus control, GFD versus control and newly diagnosed versus GFD. Of these, miR-194-5p had an increased level in the newly diagnosed group versus control, very close to a significant value (*p* = 0.0510). In addition, in the same compared groups, miR-449a and miR-638 were overexpressed and miR-192-5p was underexpressed, without statistical significance (Figure 4). When we compared the GFD group with the control group, we found that all four miRNAs had high values but of no statistical significance, miR-194-5p having a *p*-value close to the significant one of 0.0671 (Figure 5). Comparing the newly diagnosed celiac disease group with celiac disease individuals on a GFD, miR-192-5p, miR-194-5p and miR-449a were overexpressed in those on a GFD, in contrast to miR-638, which showed higher values in the newly diagnosed group, but without reaching significant difference (*p* > 0.05) (Figure 6).

### 3.4. Networks

**Figure 7:**
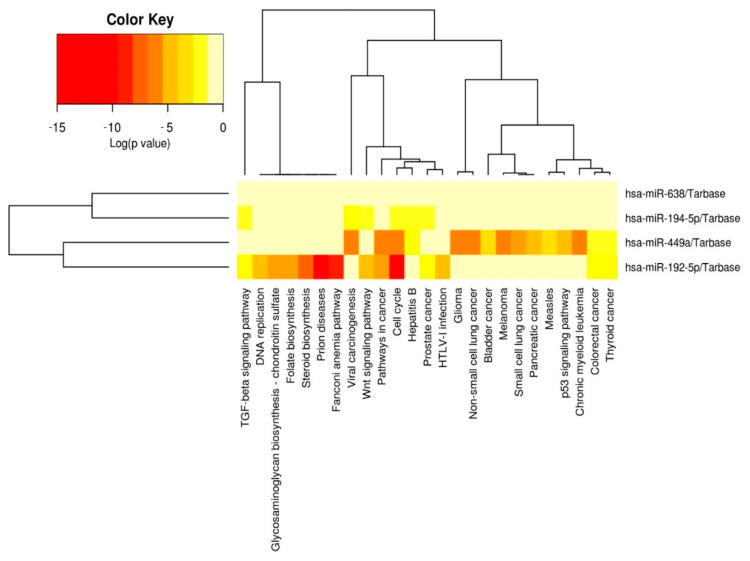
Heatmap generated using DIANA-miRPath v3.0 (http://snf-515788.vm.okeanos.grnet.gr (accessed on 5 May 2021)), showing the association with biological processes related to the evaluated miRNAs. Abbreviations: TGF, transforming growth factor; HTLV-1, human T lymphotropic virus type 1.

Figure 7 is the heatmap representation of the significant pathways targeted by the evaluated altered miRNAs signatures (miR-192-5p, miR-194-5p, miR-449a and miR-638) obtained by KEGG pathway enrichment analysis using the DIANA Tools miRPath instrument; this demonstrated significant functional activity, particularly in the case of miR-449a and miR-192-5p.

### 3.5. miRNet

**Figure 8:**
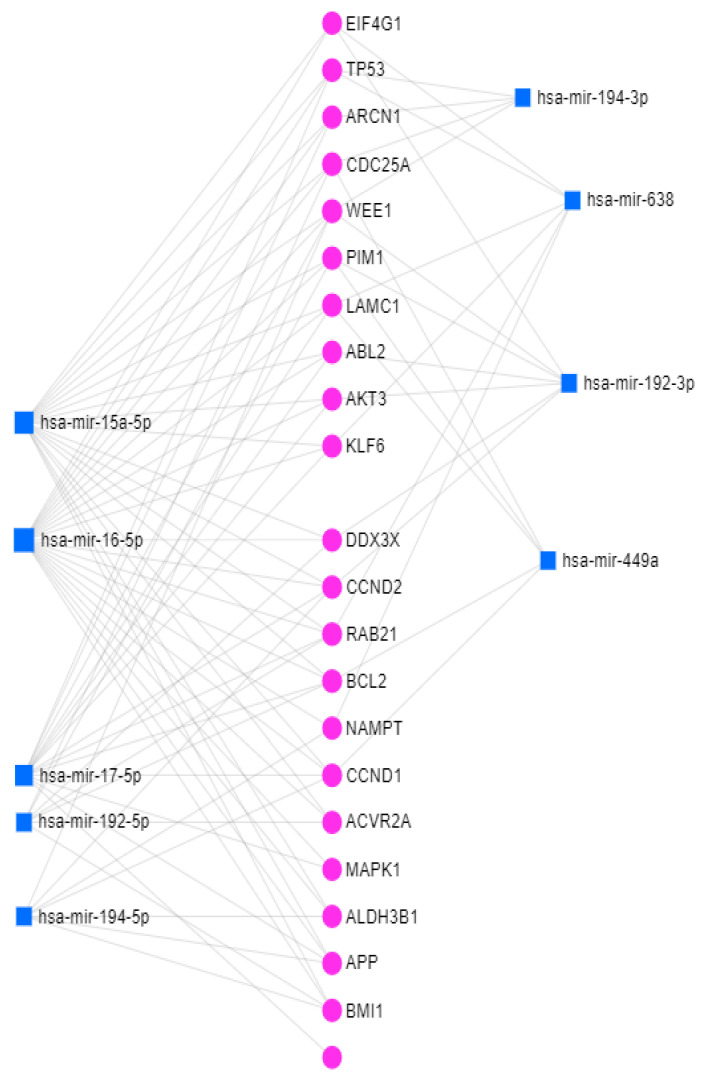
miRNA–mRNA interconnection network generated using miRNet. Abbreviations: *EIF4G1,* eukaryotic translation initiation factor 4 gamma 1; *TP53*, tumor protein p53; *ARCN1,* archain 1; *CDC25A,* cell division cycle 25A; *WEE1, WEE1 G2* checkpoint kinase; *PIM1*, pim-1 proto-oncogene, serine/threonine kinase; *ABL2*, *ABL* proto-oncogene 2, non-receptor tyrosine kinase; *KLF6*, kruppel like factor 6; *DDX3X*, DEAD-box helicase 3 X-linked; *RAB21*, *RAB21*, member RAS oncogene family; *BCL2*, *BCL2* apoptosis regulator; *NAMPT*, nicotinamide phosphoribosyltransferase; *ACVR2A*, activin A receptor type 2A; *ALDH3B1*, aldehyde dehydrogenase 3 family member B1; *APP*, amyloid beta precursor protein; *BMI1*, *BMI1* proto-oncogene, polycomb ring finger.

Altered miRNAs were able to target the gene network, revealing important interactions. Figure 8 highlights an integrative analysis of altered miRNAs and mRNAs revealing the interconnections of miR-194-5p and miR-449a with *CCND1*. 

## 4. Discussion

Celiac disease is a multifaceted medical condition in which the pathophysiological mechanisms involved have not been very well identified. As previously described, most of the genes and circulating miRNAs had elevated values in patients with CD compared to the control group. Regarding gene expression, it turned out that of those evaluated (*WNT3, WNT11, TNFα, MAPK1, AKT3, PIK3CA* and *CCND1*), *TNFα, MAPK1* and *CCND1* were statistically significant only in the group of newly diagnosed individuals compared to the control group. Of the miRNAs tested (miR-192-5p, miR-194-5p, miR-449a and miR-638), miR-194-5p was at the limit in the group of newly diagnosed CD individuals compared with controls, without reaching statistical significance (*p* = 0.0510). miR-194-5p also had elevated values close to the significant *p*-value in the GFD group compared to the control group (*p* = 0.0671).

As far as we know, in most studies, circulating miRNAs have been evaluated in pediatric CD patients. In their study on pediatric patients with CD, Buoli Comani et al. showed lower plasma miR-192-5p and miR-31-5p expression in CD patients at diagnosis than in controls. The expression of miR-192-5p, miR-31-5p and miR-21-5p in the plasma of CD patients at diagnosis and controls was conforming to duodenal histopathology. Plasma miR-192-5p was also low in patients with CD on GFD compared to controls [7]. Another study performed on children with CD by Amr et al. analyzed the serum expression of miR-21 and miR-31. They found that there was no significant difference between CD patients on a GFD and controls, in contrast to those recently diagnosed who had increased miR-21 and decreased miR-31 plasma expression compared with controls [21]. Regarding circulating miRNAs in adult patients with CD, Bascuñán et al. conducted a study on 30 patients with CD, 10 at diagnosis and 20 on a GFD for at least 1 year, where they assessed the expression of miR-21, miR-125b, miR-146a and miR-155 in peripheral blood mononuclear cells, monocytes and plasma [22]. Referring to these studies of miR-192-5p, in our study, we also obtained lower blood values in the newly diagnosed versus control groups, even if no statistically significant value was reached. In the GFD group, contrary to the other studies mentioned, we obtained higher values than those of the control group.

Vaira et al. studied the profile of miRNAs expression on the duodenal mucosa in CD patients with different clinical phenotypes and observed a significant deregulation of miR-31-5p, miR-192-3p, miR-194-5p, miR-551a, miR-551b-5p, miR-638 and miR-1290 compared to controls. There was also an increase of miR-638 expression in CD patients with anemia compared with those with CD with classic symptoms [23]. In our groups, miR-194-5p and miR-638 showed increased expression in the newly diagnosed and GFD groups versus controls.

In human embryonic kidney 293 cells (HEK-293 cells), notch receptor 1 *(NOTCH1*) and kruppel like factor 4 *(KLF4)* decreased by the high level of miR-449a. *NOTCH1, KLF4* and goblet cell counts were reduced in the intestinal mucosa of children with active CD and on a GFD compared to controls. On the other hand, a greater staining of nuclear beta-catenin and Ki67 was noted as signs of WNT activation and proliferation in CD patients than in controls [24]. Making a parallel with our study, we obtained higher miR-449a values both in the newly diagnosed group and in the GFD group than in the control group.

In animal studies, McKenna et al. identified 453 miRNAs families in epithelial cells of celiac patients and mmumiR-192 was the most expressed compared with controls. Their research study has shown an increase in crypt apoptosis in the jejunum and colon and an impairment of the intestinal barrier function, leading to severe intestinal inflammation [25].

Elevated serum *TNFα* levels at diagnosis have been reported by Street et al. compared to the controls and the values returned to normal under the gluten-free diet [26]. A significant increase in serum *TNFα* was found in patients with CD at diagnosis compared to controls [27]. Studies have been published on the effects of *TNF* in disturbing the permeability of the intestinal barrier and the tight junction [28]. Manavalan et al. reported significantly increased values of *TNFα* on dendritic cells (DC) derived from monocytes from healthy donors that were stimulated in vitro by gliadin [29]. Other studies found elevated *TNFα* levels both in jejunal biopsy and in serum in CD patients [30,31]. Marafini et al. observed statistically significant elevated values of *TNFα* in duodenal biopsies, especially in the active form of disease, compared to patients with CD on a gluten-free diet or controls [11]. *TNFα* overexpression was also noticed, in our case, in both groups of patients with CD versus control, having statistical significance in the newly diagnosed group.

Previous studies have shown the importance of Wnt signaling in the proliferation and differentiation of the gastrointestinal epithelium. The different effects of Wnt signaling in several cell types were also due to its location along the crypt/villus axis. Wnt signaling is important in the development of chronic inflammatory bowel disease and bowel cancers [32,33,34]. Gregorieff et al. described elevated levels of *WNT3* in crypt epithelial cells [34]. *WNT3* and *WNT11* were mentioned by Sato et al. among the most expressed genes in Paneth cells [35]. In our study, *WNT3* and *WNT11* values were also high in the blood of the two groups of patients with CD versus control.

There are studies showing the activation of epidermal growth factor receptor *(EGFR)–MAPK1* in CD cells [36,37,38]. In a study on PBMC in patients with CD on a GFD for at least 2 years versus controls, an increased expression of *MAPK1* was observed in those with CD [38]. In addition, another study on PBMC mentioned the aberrant activation of the MAP kinase pathway in patients with active CD [39]. Yohannes et al. described upregulation of the MAPK pathway in the PBMC of CD patients exposed to gluten and untreated [40]. Comparing with our data, we described a significantly increased expression of *MAPK1* in newly diagnosed patients versus control. Increased values were also observed in the GFD group versus the control group.

The role of *Akt* in the control of *IL-21* was evaluated by Akt phosphorylation; an increased expression of it was observed in the biopsies of CD patients compared to controls [41]. Dysregulation of the PI3K/Akt pathway is described as a link between autoimmune, malformative and neoplastic disorders [42]. Martini et al. found an upregulation of the PI3K–AKT signaling pathway and that the *AKT* gene was activated by phosphorylation. Thus, *AKT* activation stimulates metabolism, cell proliferation and intestinal size for gluten-consuming larvae [43]. In our study, the values of *AKT3* were difficult to differentiate between the newly diagnosed and control group and, in the GFD versus control groups, the elevated values did not reach statistical significance.

*PIK3CA* amplification and mutations have been poorly described in small bowel adenocarcinoma, but there were insufficient data to explain their impact on the disease [44].

Garrote et al. reported that *CCND1* had low values in the intestinal mucosa of active patients with CD [45] as opposed to our increased blood results in patients with CD, having statistical significance in those newly diagnosed compared to controls.

The study of proteomics in CD was performed in order to highlight proteins or changes in protein expression, thus discovering a biomarker using various techniques. Zamanian et al. selected articles on proteomics data in CD and, based on the study of gene expression of their articles, they extracted 31 candidate proteins. For the analysis of differentially linked protein networks, they used Cytoscape 3.3, MCODE and ClueGO [10]. Banaganapalli et al. compared the gene expression profiles of duodenal tissue samples of celiac patients at diagnosis and after 2 years of gluten-free diet. They aimed to identify protein interactions and molecular pathways involving several differentially expressed genes (DEG) and to present a broader picture of changes in gene expression important for CD pathogenesis [9].

Studies on the links between miRNA function and autoimmunity were based on the important roles of miRNAs in regulating the immune response and in the development of immune cells. Studies on cell cultures and animals showed that regulation of miRNA helped to prevent autoimmunity and maintain normal immune function. On the other hand, it is not clear whether miRNA dysregulation has any implications for the pathogenesis of autoimmune diseases in humans. There are studies that have described some of their implications in autoimmune diseases. The role of miRNAs as potential targets in the treatment and prevention of certain autoimmune diseases has been speculated [46].

Another study described the role of miRNAs in cell development and differentiation and the release of inflammatory cytokines. What is certain is the role of miRNAs in regulating innate immune responses, particularly macrophages and granulocytes. Regarding the involvement of miRNAs in adaptive immune responses, abnormal expression has been observed in patients with autoimmune diseases. There are studies that also speculated on their role as therapeutic targets and biomarkers in allergic diseases [47].

Celiac disease can sometimes take subclinical/silent forms and may be associated with other immunological and non-immunological diseases. Although atopy has been frequently associated with CD, the prevalence of celiac disease in atopic individuals has not been clearly established. In the study by Zauli et al., a 1% prevalence of celiac disease in atopic individuals has been described. Therefore, the authors mentioned that atopy should be considered a risky condition and atopic patients should be routinely examined by testing for specific autoantibodies [48].

In a study by Granito et al., in addition to IgA anti-transglutaminase and IgA anti-endomysium antibodies, other antibodies, such as IgA antibodies against actin filaments (AAA), antimicrofilament IgA antibodies (IgA anti MF), tubular/glomerular pattern of anti-smooth muscle antibodies (SMA-T/G), tubular/glomerular patterns of anti-smooth muscle IgA antibodies (IgA SMA-T/G) were described. They have been observed in patients with moderate or severe atrophy, showing that they correlate with the degree of mucosal damage. Their discovery is closely associated with the flat mucosa and is predictive of severe gluten-sensitive enteropathy. Due to their low overall sensitivity, the authors concluded that they could not replace anti-transglutaminase and anti-endomysium antibodies in the diagnosis of CD but could supplement them in the follow-up of severe celiac disease [49].

As described in Table 1, of the patients on a GFD, nine were with Marsh–Oberhuber 3a histology, eight with Marsh–Oberhuber 3b and three with Marsh–Oberhuber 3c. Regarding the gluten-free diet, the anamnesis of these patients showed that, of the nine patients with Marsh–Oberhuber 3a, seven said that they followed the diet strictly and two that they partially followed it; of the eight patients with Marsh–Oberhuber 3b, two said they strictly followed the diet and the other six only partially; of the three patients with Marsh–Oberhuber 3c, one said that he strictly followed the diet and the other two only partially.

Table 1 also shows that approximately 60% of patients with celiac disease had negative antibodies. Of the 19 (57.6%) on a GFD, 8 were negative from diagnosis. A role in the pathogenesis of seronegative celiac disease is, apparently, the impossibility of the passage of antibodies (IgA anti-transglutaminase) from the intestine into circulation, caused by the fact that they bind to tranglutaminase 2, with which they form immunocomplex deposits [50]. There are studies that confirm this hypothesis [51,52,53,54]. Another explanation of seronegativity in patients with CD is the immaturity of the immune system, with the association of immunoglobulin deficiencies such as IgA deficiency or common variable immunodeficiency [50].

Seronegative celiac disease is a form of celiac disease that is still unclear in many ways, representing a challenge for clinicians, especially in terms of differential diagnosis. The prevalence of seronegative celiac disease varies, in the literature, from 1.03 to 28% [55,56]. There are studies that present series of cases with seronegative celiac disease. An example is the study by Jorje Bernardo et al., performed on a number of 12 seronegative patients with digestive and extra-digestive manifestations of CD [57]. Another example is the study by Kotze et al., performed on 10 seronegative patients with clinical manifestations suggestive of celiac disease [56]. In the study conducted by Volta et al., of 31 patients with seronegative villous atrophy, 14 (45%) had seronegative celiac disease; celiac disease was the most common cause of seronegative villous atrophy in their study [58]. In a prospective study conducted over a period of 15 years, Aziz et al. found that, of the 200 patients with negative antibodies and villous atrophy, 31% had seronegative celiac disease [59].

Another aspect in our patients was that a large number of them had extra-intestinal manifestations (60% in the newly diagnosed group and almost 50% in the GFD group). In the literature, there are studies on the pathogenesis of extra-intestinal manifestations, incompletely elucidated. Two main mechanisms seem to be involved, one related to mucosal damage with consecutive malabsorption and the second represented by the autoimmune response. The hypothesis of autoimmune phenomena requires additional evidence. In addition to tissue transglutaminase 2 (TG2), the major autoantigen involved in CD, there are other autoantigens described. The role of anti-TG2 autoantibodies in pathogenesis has not been clearly demonstrated. They are known to bind to various epitopes, including the enzyme core, and may then be related to TG2 bioactivity. A research study on extra intestinal manifestations describes implications of the autoantigens transglutaminase 3, transglutaminase 6, gangliosides, synapsin I and collagen [60,61,62]. Extra-intestinal manifestations were described many years ago, but their prevalence has not been clearly specified. This is also due to variable definitions and important age-related variations in the onset of symptoms. There are studies that describe a prevalence of extra-intestinal manifestations around 60% in both adults and children [63]. In a study by Jericho et al., the authors obtained a similar percentage of extra-digestive manifestations in adults with CD, namely, 62% [64]. In another study by Sansota et al., it was found that patients with extra-intestinal manifestations had a lower rate of improvement than those with digestive manifestations after more than 2 years of GFD. It was also found that, in adults, both digestive and extra-digestive manifestations improved later than in children. Predictors of this low rate of clinical improvement that the authors found were female gender, long duration of symptoms and non-adherence to the GFD [65]. Even with a strict diet, certain extra-intestinal manifestations may only partially improve, which may indicate a much more complex etiology [63]. In patients with extra-intestinal manifestations, other autoantibodies may play a role in the mechanisms of the disease and in the diagnosis [62]. The distinct appearance of serological markers is consistent with the different pathogenetic mechanism of the manifestations of celiac disease [63].

The fact that, in our study, only a few genes reached a statistical significance and that some miRNAs were close to reaching statistical significance, denotes certain limitations in terms of the small number of patients and the need for additional studies.

## 5. Conclusions

In conclusion, our study offers a new perspective on the pathogenesis of celiac disease by describing the expression of selected circulating miRNAs and genes and by analyzing miRNA–mRNA networks. Understanding the molecular mechanisms and pathways involved could lead to the discovery of new biomarkers and targeted therapy. Analysis of the interconnection between miR-194-5p and miR-449a with *CCND1* may provide future research opportunities and open new perspectives in personalized and targeted therapeutic strategies.

## Figures and Tables

**Table 1:** General characteristics of CD patients and controls.

Characteristics	Subjects		
	Newly Diagnosed (*n* = 15)	GFD (*n* = 33)	Control (*n* = 10)
Sex, *n;* females (%)	13 (86.6)	27 (81.8)	6 (60)
Mean age, years; ± S.D.	42.3 ± 12.3	45 ± 14.9	51.3 ± 13.2
Gastrointestinal manifestations, %	40	51.5	0
GFD duration, years; mean ± S.D.	0	5.4 ± 6.2	0
Family history for:			
CD, %	0	15.1	0
Other autoimmune diseases, %	20	15.1	0
Marsh–Oberhuber stage, *n* (%)	T3a = 4 (26.6) T3b = 6 (40) T3c = 5 (33,3)	T0 = 8 (24.2) T1 = 1 (3.03) T2 = 4 (12.1) T3a = 9 (27.2) T3b = 8 (24.2) T3c = 3 (9.09)	normal duodenal histology
Positive IgA tTG and/or IgA EMA, *n* (%)	6 (40)	14 (42.4)	0

Abbreviations: n, number; S.D., standard deviation;

## Data Availability

The data presented in this study are available on reasonable request from the corresponding author.
